# Maternal, Fetal, and Neonatal Outcomes of Gestation in Women with and Without *Brucella* Infection

**DOI:** 10.34172/jrhs.2023.110

**Published:** 2023-03-30

**Authors:** Mohammad Mahdi Majzoobi, Roya Teimori, Shahla Nouri, Manoochehr Karami, Mile Bosilkovski, Ali Saadatmand

**Affiliations:** ^1^Infectious Diseases Research Center, Hamadan University of Medical Sciences, Hamadan, Iran; ^2^Department of Infectious Diseases, School of Medicine, Hamadan University of Medical Sciences, Hamadan, Iran; ^3^Department of Family Health, Hamadan University of Medical Sciences, Hamadan, Iran; ^4^Department of Epidemiology, School of Public Health & Safety, Shahid Beheshti University of Medical Sciences, Tehran, Iran; ^5^University Clinic for Infectious Diseases and Febrile Conditions, Medical Faculty University “Ss Cyrilus and Methodius”, Skopje, Republic of North Macedonia

**Keywords:** Brucellosis, Pregnancy, Abortion, Low birth weight

## Abstract

**Background:** Maternal, fetal, and neonatal complications of brucellosis in pregnant women are probably higher than those in the general population. This comparative study aimed to survey the mentioned complications in pregnant women with positive and negative *Brucella* serologic tests.

**Study Design:** This is a prospective cohort study.

**Methods: ** In this study, 2160 pregnant women residing in the rural area of Hamadan province were screened for *Brucella* infection by agglutination test. Then, 106 (4.90%) pregnant women with a positive test (exposed group) were compared with 210 subjects (non-exposed group) who were randomly selected from more than 2000 pregnant women with a negative serological test in terms of maternal, fetal, and neonatal outcomes from October 2018 to March 2020. Data were analyzed by SPSS 20 software at a 95% confidence level.

**Results:** The mean age of mothers in both exposed and unexposed groups was 27.84±6.13 and 38.71±6.85 years, respectively. Past medical history of brucellosis, animal contact, and the consumption of unpasteurized dairy products were reported to be 14 (13.2%), 63 (59.4%), and 82 (77.4%), respectively, in the exposed group. The mentioned measures were 3 (1.5%), 109 (51.9%), and 54 (26.9%) in the unexposed group, respectively. Among exposed and unexposed groups, the incidence of abortion was 9 (8.6%) and 5 (2.4%) with *P*=0.005, intrauterine fetal death was 2 (1.9%) and zero with *P*=0.211, low birth weight was 10 (10.6%) and 7 (3.4%) with *P*=0.012, and premature birth was 15 (15.2%) and 18 (8.8%) with *P*=0.066, respectively.

**Conclusion:** Brucella infection in pregnant women appears to be associated with the risk of miscarriage, low birth weight, and premature birth.

## Background

 Brucellosis is one of the most common zoonotic diseases worldwide and is an important public health issue in some regions, including Asia and Mediterranean countries.^1^ Brucellosis is transmitted to humans through direct contact with infected animals or by consuming their dairy products. Livestock farmers, slaughterhouse workers, and veterinarians are at risk for brucellosis.^2^ Clinical symptoms of the disease include generalized pain, arthralgia, headache, fever, arthritis, and to a lesser extent, spondylitis and orchitis.^3^

 Most parts of Iran are endemic for brucellosis, and due to the close collaboration between men and women in livestock and agricultural activities, brucellosis is also common in women. In 2009, the incidence of brucellosis in Western Iran was 59.31 per 100 000 (34.9% in women and 65.1% in men), and approximately 95.2% of human brucellosis cases belonged to rural areas.^4,5^ Further, healthy individuals practicing animal husbandry in endemic areas may have elevated Brucella antibody titers.^6^

 The seroprevalence of brucellosis during pregnancy varies across countries as 5.8%,^2^ 1%-1.8%,^7^ and 3.5%.^8^ The cumulative incidence of brucellosis in pregnancy varied from 0.42 to 3.3 per 1000 gestation.^7,8^ It seems that brucellosis plays a significant role in the incidence of abortion and intrauterine death compared to other bacterial infections. Maternal bacteremia, acute fever, toxemia, and disseminated intravascular coagulation are the mechanisms by which brucellosis causes spontaneous abortion and intrauterine fetal death.^7^

 A significant percentage of miscarriages have unknown causes.^9^ Numerous studies highlighted the role of infection in miscarriage, especially in the second trimester of pregnancy.^10^ Since livestock contains erythritol, *Brucella* spp. invade embryonic tissue and cause abortion. Although human tissue lacks erythritol, and *Brucella* antibodies in the amniotic fluid reduce the risk of pregnancy complications caused by the bacteria, *Brucella* spp. can enter the uterine cavity.^9^ Generally, abortion mostly occurs in the second trimester of pregnancy, and intrauterine bleeding and fever are the most prevalent signs.^11,12^ The rate of spontaneous abortion in women with brucellosis was 6.9%,^13^ 11.8%,^14^ 18.3%,^15^ and 19%^16^ in Kuwait, Iran, Rwanda, and Nigeria, respectively. Most studies emphasized the effect of treatment on controlling brucellosis complications, especially abortion in pregnancy.^17^ Moreover, combination therapy with rifampin and cotrimoxazole for six weeks is the most commonly administered treatment.^1,11,17^ The prevalence of preterm delivery in women with brucellosis, compared to those without it, was reported to be 17.9% vs. 2.5%.^18^ Additionally, the rate of intrauterine fetal death in pregnant women with brucellosis varied across studies from 10% to 20.6%.^19^

 The present study aimed at determining the prevalence of seropositive pregnant mothers with any positive titer of Wright and 2-mercaptoethanol (2ME) and comparing maternal, fetal, and neonatal outcomes of pregnancy between seropositive and seronegative pregnant women.

## Methods

 In the present prospective cohort study, 106 pregnant women seropositive for *Brucella* (exposed group) and 210 counterparts seronegative for *Brucella* (unexposed group) were selected from Malayer, Famenin, and Kaboudar-Ahang cities, Hamadan Province, Western Iran. Subsequently, the maternal, fetal, and neonatal outcomes of the disease in pregnant women were followed up and compared from Oct 2018 to March 2020.

 The appropriate sample size was determined using the Kelsey sample size formula for cohort studies. To enroll 106 subjects exposed to *Brucella* spp., 2160 pregnant women were screened by the Wright and 2ME tests. The unexposed group was randomly selected from seronegative pregnant women in the mentioned cities. Sampling was performed using convenience and consecutive sampling methods.

###  Data Collection

 Pregnant women living in rural areas of Malayer, Famenin, and Kaboudar-Ahang were screened for *Brucella* infection by the Wright test in the 6th week of gestation on the first pregnancy care, along with routine medical tests after obtaining written informed consent. Those who were seropositive for *Brucella* based on the Write test titer underwent 2ME. Seropositive subjects (with and without clinical signs) were enrolled in the exposed group, and the controls (approximately twice the exposed group) were randomly selected from three cities. Then, both groups were studied in terms of pregnancy outcomes.

 Pregnant women with chronic diseases with the potential to affect the pregnancy product such as lupus erythematous and pregnant women who missed the pregnancy product due to eclampsia or preeclampsia were excluded from the study.

 The experiments were performed in the central medical laboratories of Malayer, Kaboudar-Ahang, and Famenin, and the kits and method titration were matched in the reference laboratory of Brucellosis Research Center, Hamadan University of Medical Sciences. The kits were purchased from the Pasteur Institute of Iran. The concurrent Wright titer of ≥ 1.80 and 2ME titer of ≥ 1.40 were considered positive to detect patients in the exposed group based on the Iranian National guidelines for Brucellosis Control.^20^

 Other data (e.g., age, place of residence, history of pregnancy, history of raw milk and unpasteurized dairy products consumption, contact with livestock, general symptoms, and history of miscarriage) were recorded for each subject using the questionnaire. The subjects diagnosed with brucellosis were followed up through health centers throughout the gestation and immediately after delivery in terms of maternal, fetal, and neonatal outcomes of the disease.

 The first, second, and third trimesters of pregnancy were defined as gestational age under 12 weeks, 12-24 weeks, and above 24 weeks, respectively. Fetal death at gestational age under 24 weeks, spontaneous abortion, and death at gestational age above 24 weeks were defined as intrauterine death.^12^ Childbirth before 38 weeks of gestation was considered preterm labor, and birth weight less than 2500 grams was regarded as a low birth weight.^10^

 After the completion of the questionnaires, data were analyzed by SPSS 20 software (SPSS Inc., Chicago, IL, USA) at a 95% confidence level. Descriptive data were expressed as frequency, percentage, mean, standard deviation, and prevalence. The relationship between brucellosis and pregnancy outcomes was evaluated using the Fisher exact and chi-square tests. Moreover, an independent *t* test was applied to compare the mean of quantitative variables in two groups. The prevalence of brucellosis was expressed as the percentage of patients with *Brucella* infection, while pregnancy outcomes were reported cumulatively for the exposed and non-exposed groups. The results of analyses were reported at a 95% confidence interval.

###  Ethical Considerations

 The study protocol was approved by the Ethics Committee of Hamadan University of Medical Sciences (Ethical code: IR.UMSHA.REC.1397.667). The subjects were charged for enrollment in the study and laboratory tests.

## Results

 Totally, 106 diagnosed seropositive persons with 210 seronegative subjects who had been randomly selected out of 2160 pregnant women, based on concurrent Wright and 2ME titers, were compared in terms of maternal, fetal, and neonatal outcomes.

 Based on Table 1, no significant difference was observed in the mean age of the positive and negative groups. The mean ± standard deviation age in the positive and negative groups was 27.84 ± 6.13 and 38.71 ± 6.8, respectively. Furthermore, the prevalence of brucellosis history in the positive was significantly higher than that in the negative group (14 (13.2%) versus 3 (1.5%); *P* value = 0.001). Moreover, the prevalence of abortion and low birth weight were significantly higher in seropositive subjects than in controls (*P*< 0.05).

**Table 1:** Baseline characteristics of studied participants regarding *Brucella* infection

**Continuous variables**	**Positive**		**Negative**		***P*** ** value**
	**Mean**	**SD**	**Mean**	**SD**	
Age	27.84	6.13	38.71	6.85	0.972
**Categorical variables**	**Number**	**Percent**	**Number**	**Percent**	
History of brucellosis	14	13.2	3	1.5	0.001
Livestock contact	63	59.4	109	51.9	0.057
The consumption of unpasteurized dairy products	82	77.4	54	26.9	0.491

*Note.* SD: Standard deviation.

 According to Table 2, based on the Iranian National Guidelines for Brucellosis Control, out of 106 pregnant women with positive tubal agglutination test, 38 (35.8%) were identified as patients with brucellosis (concurrent wright ≥ 1.80 and 2ME ≥ 1.40), and all of them reported a history of contact with livestock and the consumption of unpasteurized dairy products. Additionally, the most common clinical symptoms in these patients were fatigue (31.5%), joint pain (23.7%), low back pain (18.4%), headache (13.1%), and fever (10.5%).

**Table 2:** Frequency distribution of 2ME titers according to wright titers among positive serology pregnant women

**Wright**	**2ME**									
	**Negative**		**1/20**		**1/40**		**≤1/80**		**Total**	
	**Number**	**Percent**	**Number**	**Percent**	**Number**	**Percent**	**Number**	**Percent**	**Number**	**Percent**
1/20	10	41.7	15	58.3	0	0	0	0	25	100
1/40	3	8.6	26	74.3	5	17.1	0	0	34	100
1/80	2	6	7	21.2	15	45.5	9	27.3	33	100
≤ 1/160	0	0	0	0	1	7.1	13	92.9	14	100
Total	15	14.1	47	44.3	22	20.8	22	20.8	106	100

*Note.* 2ME: 2-Mercaptoethanol.

 As depicted in Table 3, the prevalence of abortion and low birth weight were significantly higher in seropositive subjects than in controls (*P*< 0.05). Although no significant differences were observed between the groups in terms of intrauterine fetal death, it was close to significant for gestational age at delivery. Moreover, the frequency of neonatal and maternal outcomes, including miscarriage, intrauterine death, low birth weight, and preterm labor in seropositive subjects had no association with Wright or 2ME titer value. There was no significant difference between the frequency of preterm labor and low birth weight in subjects with a Wright titer of below or above 1.80 as well as the presence or absence of clinical symptoms; furthermore, the number of abortions was higher in symptomatic subjects, although the difference was insignificant (*P*= 0.070). In addition, the mean birth weight of newborn infants in both seropositive and control groups was 3190.1685 ± 548.442 and 3288.951 ± 471.330 grams, respectively (*P*= 0.117). Furthermore, the mean gestational age at the time of delivery in the seropositive and control groups was 38.74 ± 2.46 and 39.05 ± 1.32 weeks, respectively (*P*= 0.160).

**Table 3:** Maternal and neonatal outcomes in pregnant women with and without positive tube agglutination test

**Outcome**	**Positive test (n=106)**		**Negative test (n=210)**		**RR (95% CI)**	***P*** **-value**
	**Number**	**Percent**	**Number**	**Percent**		
Abortion						
No	96	91.4	205	97.6	1.00	
Yes	9	8.6	5	2.4	3.60 (1.23, 10.47)	0.005
IUFD						
No	104	98.1	210	100	1.00	
Yes	2	1.9	0	0	Unpredictable	0.211
Birth weight (g)						
≥ 2500	84	89.4	198	96.6	1.00	
< 2500	10	10.6	7	3.4	3.11 (1.22,7.93)	0.012
Gestational age (wk)						
≥ 37	84	84.8	187	91.2	1.00	
< 37	15	15.2	18	8.8	1.81 (0.95,3.44)	0.066

*Note.* RR: Risk ration; CI: Confidence interval; IUFD: Intrauterine fetal death.

## Discussion

 In the present study, 2160 pregnant women were screened for *Brucella* infection by the Wright test, of which 106 (4.88%) were seropositive. Of the seropositive subjects, 35.8% had concurrent titers of Wright ≥ 1.80 and 2ME ≥ 1.40. In a cohort study in Western Iran, 6.59% of the urban and rural population was seropositive based on the Wright test.^21^ In a study on butchers and slaughterhouse workers, 13.3% were seropositive based on the Wright test.^22^ The lower prevalence reported in the present study may be due to the study population, which included only women with less direct contact with livestock than men.

 In the present study, 22 patients (20.75% of 106 seropositive pregnant women and 46.80% of 47 pregnant women with a Wright titer of ≥ 1.80) were symptomatic, and fatigue, arthralgia, and back pain were the most prevalent symptoms. In the study by Mamani,^22^ 20.6% of the patients had clinical symptoms, and the most common ones were myalgia, fatigue, and back pain. Kurdoglu et al,^7^ Inan et al,^8^ and Liu et al,^23^ reported night sweats, anorexia, and fever as common symptoms, in addition to the mentioned complaints.

 In the present study, the frequency of abortion was 9.4%, intrauterine death 1.9%, low birth-weight 10.6%, and preterm birth 16.0% in seropositive subjects. In the study by Liu et al, 31.3% of subjects experienced preterm delivery, 37.5% abortion, and 9.8% intrauterine death.^23^ In the study by Kurdoglu et al^7^ on the complication of brucellosis in 29 pregnant women, seven (24.11%) subjects had a spontaneous abortion, one (3.45%) intrauterine death, and two (6.39%) premature delivery. The rate of abortion and intrauterine death was lower, and preterm delivery in the present study was higher than that in Kurdoglu et al’s study. Further, in a case-control study by Elshamy and Ahmed^11^ on the impact of maternal brucellosis on the gestational product, the incidence of spontaneous abortion was 27.7%, intrauterine death 12.72%, and preterm delivery 10.9%.

 The higher prevalence of the complications in the above-mentioned studies might be due to the study population, which consisted of only patients with known *Brucella* disease, while the present study only recruited seropositive pregnant women detected through screening, and a significant number of them did not exhibit clinical manifestation.

 According to Kledmanee et al, brucellosis increased the prevalence of abortion by 1.8 times^24^ which was a little higher than that of the present study, which was 1.23 times. In a review study by Bosilkovski et al on the outcomes of human brucellosis during pregnancy, the most frequent complications were abortion (2.5%-54.5%), intrauterine fetal death (0-20.6%), and premature birth (1.2%-28.6%),^25^ which were consistent with results of the current study.

 Based on the findings of the present study, no significant relationship was found between concurrent serum titers of ≥ 1.80 and ≥ 1.160 and pregnancy outcomes. However, Elshamy and Ahmed detected a significant difference in the incidence of abortion between pregnant women with a *Brucella* antibody titer of > 1.160 and < 1.160.^11^

 In a study by Hasanjani Roushan et al, 55% of women with brucellosis experienced spontaneous abortion in the first trimester of pregnancy, but no significant relationship was found between serum agglutination titer and abortion.^26^ According to the findings of the study by Nassaji et al, no significant relationship was observed between brucellosis and antibody titers.^27^ In the study by Khan et al, the prevalence of spontaneous abortion and intrauterine death was 43% and 2%, respectively, in pregnant women with brucellosis, and no correlation was found between higher titers of the Wright test and bacteremia.^28^ In the current study, maternal and neonatal complications were higher in pregnant women with positive Wright test; however, no correlation was found between the Wright test titer and clinical symptoms. It may reflect the fact that asymptomatic infection can increase the chances of complications in a healthy population. In line with our study, in a study by Vilchez et al, spontaneous abortion, preterm births, intrauterine fetal death, and low birth weight were observed in 12.8%, 13.9%, 1.8%, and 8.1% of subjects, respectively.^29^

 The treatment of brucellosis in pregnant women with safe drugs can reduce the risk of maternal and neonatal complications.^30^ In the present study, pregnant women were screened in early pregnancy, and symptomatic cases with Wright and 2ME positivity were treated promptly, explaining the lower rate of brucellosis complications during pregnancy compared with some other studies.

**Highlights**

- *Brucella* infection in pregnant women is associated with the risk of miscarriage.
- *Brucella* infection in pregnant women is associated with the risk of low birth weight.
- *Brucella* infection in pregnant women is associated with the risk of premature birth.

## Conclusion

*Brucella* infection in pregnant women, regardless of the presence or absence of clinical symptoms and value of Wright and 2ME titer, might be associated with the risk of miscarriage, low birth weight, and preterm labor.

## Acknowledgments

 This study was approved by the Vice-chancellor of Research and Technology, Hamadan University of Medical Sciences, Hamadan, Iran, under the number 9711096778.

## Authors’ Contribution

 MMM contributed to the conception, conducting of the study, revising the draft, and approval of the final version of the manuscript. RT, SN, and AS contributed to conducting the study. MK contributed to the study analysis, and MB revised the draft and approved the final version of the manuscript.

## Competing Interests

 The authors declare no conflict of interests.
